# Effects of Algae β‐Glucan Supplementation on the Performance and Intestinal Immune System of Healthy Weaned Piglets

**DOI:** 10.1111/jpn.70077

**Published:** 2026-05-20

**Authors:** Johanna O. Buckenberger, Felizitas Gutöhrlein, Manfred Henrich, Erika Most, Rose Whelan, John K. Htoo, Robert Ringseis, Klaus Eder

**Affiliations:** ^1^ Institute of Animal Nutrition and Nutritional Physiology Justus Liebig University Giessen Giessen Germany; ^2^ Institute of Veterinary Pathology Justus Liebig University Giessen Giessen Germany; ^3^ Evonik Operations GmbH Hanau‐Wolfgang Germany; ^4^ Center for Sustainable Food Systems Justus Liebig University Giessen Giessen Germany

**Keywords:** β‐glucan, immune system, inflammation, paramylon, piglet

## Abstract

While β‐glucans from plants and yeasts have been extensively studied in weaned piglets, studies examining algae β‐glucans are scarce. Thus, the present study aimed to examine the effects of feeding algae β‐glucan in weaned piglets. Male, 21‐day‐old, weaned crossbred piglets were allotted to three experimental groups, which were fed either an adequate basal diet (Control) or the basal diet supplemented with 100 (A100) or 200 g/t (A200) of algae β‐glucan for 35 days. Performance parameters, apparent total tract digestibility of nutrients, gut morphology metrics, gut mucosa mRNA levels of most genes involved in gut function and integrity, and the number of different immune cells in the proximal jejunum did not differ across groups after 35 days. In the mid‐jejunum, the number of lymphocytes in the *lamina propria* was higher in the A200 group than in the other two groups (*p* < 0.05) after 35 days. Mucosa score for mucosa fibrosis, villous stunting and villous epithelial injury in the duodenum and the proximal and mid‐jejunum were not different across groups after 35 days. The proportion of acetate was higher, and that of valerate was lower in the colon digesta of the A200 group than in the other two groups (*p* < 0.05). While the abundance of *Prevotella* spp. was lower in the A200 group than in the other groups (*p* < 0.05), the abundance of *Bifidobacterium* spp., *Clostridium‐leptum*‐group, *Faecalibacterium prausnitzii* and *Lactobacillus* spp. in the colon digesta did not differ across groups after 35 days. In conclusion, feeding algae β‐glucan to healthy weaned piglets marginally influences gut‐associated parameters and the abundance of selected bacterial groups and the bacterial fermentation pattern in the colon digesta but has no effect on growth performance.

## 1 Introduction

Weaning of piglets in commercial production systems occurs at a very early stage of life, typically between 21 and 28 days after birth, during which the intestine, including the intestinal immune system, is not yet fully developed (Butler et al. 2000). Besides the dietary transition from liquid to solid feed, weaning involves separating piglets from their mothers and exposing them to environmental and social changes, making it a challenging event for the piglet (Carroll et al. 1998; Campbell et al. 2013). Due to these rapid changes, voluntary feed intake, as well as intestinal integrity and function, are compromised during the weaning phase (Hampson 1986; Pluske et al. 1997; Boudry et al. 2004). Impaired intestinal integrity and function in piglets manifest as intestinal inflammation, villous atrophy, crypt hyperplasia and reduced epithelial brush border activity—factors that are known to promote diarrhoea, bacterial translocation and reduced digestive and absorptive capacities of the small intestine, thereby impairing daily weight gain (Lallès et al. 2004). Since antibiotic growth promoters, previously used to address these weaning‐associated challenges, have been banned in livestock feed in the EU, alternative strategies must be applied to mitigate weaning‐associated stress.

Nutritional interventions, in particular, are viable options because diet plays a pivotal role in gut microbial colonisation and development, thus representing a compelling target for modulating gut microbiota and immune function. Convincing evidence has demonstrated that supplementing the diet of weaned piglets with plant‐derived polysaccharides, including β‐glucans, increases the abundance and colonisation of beneficial intestinal microbes, reduces the incidence of gut infections and diarrhoea and thereby improves gut health and growth performance (O'Doherty et al. 2023; Barducci et al. 2024; Huang et al. 2024). β‐Glucans are polysaccharides consisting of glucose monomers linked by β‐glycosidic bonds, and they occur naturally in plants, yeasts, fungi, algae, protozoa and bacteria (Dalmo and Bøgwald 2008; Volman et al. 2008; Wismar et al. 2010). As pathogen‐associated molecular patterns, β‐glucans are recognised by specific receptors, such as Toll‐like receptors (TLR), expressed by cells in the gut lining, for example, intestinal epithelial cells (IEC) and gut‐associated immune cells (Goodridge et al. 2011). Consequently, β‐glucans exhibit immunomodulatory activities (Volman et al. 2008; Goodridge et al. 2009; Song et al. 2014). Beyond this, β‐glucans serve as fermentation substrates for gut microbes, thereby exerting prebiotic effects on certain bacterial groups and modulating the intestinal microbiota composition (Huang et al. 2024). Because β‐glucans vary significantly in structure (e.g., type and degree of branching), molecular weight, solubility and fermentability, the biological effects of diverse β‐glucan sources may vary substantially (Dalmo and Bøgwald 2008; Volman et al. 2008). While numerous studies have investigated β‐glucans from plants and yeasts in weaned piglets, studies examining β‐glucans derived from algae are scarce. β‐Glucans from the microalga *Euglena gracilis* consist primarily of paramylon, a linear β‐glucan composed exclusively of β‐1,3‐glycosidic linkages and stored in the cytoplasm. It accounts for approximately 50% of the microalgae's dry mass (Tanaka et al. 2017; Barsanti et al. 2022).

Given this background, the present study aimed to examine the effects of dietary supplementation with an algal β‐glucan product on growth performance, nutrient digestibility, gut morphology, gut integrity parameters, the gut immune system and the abundance of selected bacterial groups in weaned piglets. Critical parameters of gut integrity included the intestinal expression of selected tight‐junction (TJ) proteins, which, together with adherens junctions and desmosomes, are responsible for sealing adjacent epithelial cells near the luminal surface to prevent the paracellular passage of luminal antigens (Alizadeh et al. 2022). Additionally, gut integrity was assessed through mucosa scoring for the occurrence of mucosal fibrosis, villous stunting and epithelial injury in the piglets' small intestine. The effect on the gut immune system was evaluated by measuring the expression of proinflammatory genes, known to be produced by IEC and gut‐associated immune cells in response to microbial stimuli or tissue damage (Stadnyk 2002; Burkey et al. 2009; Peterson and Artis 2014), and by counting various gut‐associated immune cells. These included intraepithelial lymphocytes (IEL), which secrete proinflammatory mediators in response to microbial stimuli and luminal antigens (Stadnyk 2002; Burkey et al. 2009; Neurath 2014), mucin‐producing goblet cells, as well as lymphocytes, eosinophils and neutrophils in the *lamina propria*.

## 2 Methods

### 2.1 Animals and Experimental Design

The feeding trial with weaned piglets was approved by the Animal Welfare Officer of the Justus Liebig University Giessen (approval no. JLU 602_M). All experimental procedures described followed established guidelines for the care and handling of laboratory animals. A 5‐week feeding trial was performed with 66, 21‐day‐old, male weaned crossbred piglets ([DE × DL] × Pietrain) with an average body weight (BW) of 6.68 ± 0.99 kg (mean ± SD). The piglets were housed in an air‐conditioned barn (ambient temperature decreasing from 27.5°C to 25°C during the trial, relative humidity 50%, 12 h light–dark cycle) with 11 boxes with three piglets each, and the experiment was performed in two consecutive runs (blocks) with 22 boxes in total. The piglets were allotted to three experimental groups of similar mean BW, which were fed three different diets, which met the nutrient requirements of growing pigs (German Society for Nutrition Physiology GfE 2006), in a 2‐phase feeding system (Phase I: d 1–16, Phase II: d 17–35). The three diets were either the basal diet only (control group) (*n* = 8 boxes), whose composition is shown in Table 1, or the basal diet supplemented with either 100 g/t (A100 group) or 200 g/t (A200 group) (each *n* = 7 boxes) of an algal β‐glucan product from the microalga *E. gracilis* provided by Evonik Operations GmbH (Hanau, Germany). This algae product contains at least 45% β‐glucans. The product is produced by fermentation of a natural, non‐genetically modified microalga strain in a controlled, sterile fermentation system. Throughout the trial, the piglets had free access to the diets and to drinking water via nipple drinkers. The BW and feed consumption were recorded weekly.

**Table 1:** Ingredient and nutrient composition of the basal diets.^a^

	Phase I	Phase II
Ingredients, % as‐is basis		
Corn	30.32	42.75
Wheat	20.00	20.00
Soybean meal, 44% CP	31.99	30.86
Whey powder	9.60	—
Soybean oil	3.39	1.75
Premix^b^	3.00	3.00
L‐Lys‐HCl	0.51	0.52
MetAMINO (99% DL‐methionine)	0.27	0.23
ThreAMINO (98.5% L‐threonine)	0.21	0.21
ValAMINO (98% L‐valine)	0.12	0.10
TrypAMINO (98% L‐tryptophan)	0.08	0.08
TiO_2_	0.50	0.50
Chemical composition, % dry matter^c^		
Dry matter	88.7	88.8
Crude ash	7.06	6.62
Crude protein	23.5	21.8
Crude fibre	4.75	4.96
Crude fat	6.20	4.95
ME (MJ/kg)	15.7	15.5
Standardised ileal digestible (SID) lysine	1.52	1.42
SID methionine + cysteine	0.92	0.87
SID threonine	0.97	0.91
SID tryptophan	0.34	0.32

^a^ Phase I diet was fed from a piglet BW of 6.68 ± 0.99 to 11.15 ± 1.34 kg, Phase II diet from 11.15 ± 1.34 to 24.25 ± 2.54 kg of piglet BW. For preparation of the experimental diets, the algal β‐glucan supplement was added at the expense of wheat.

^b^ The premix contained per kg diet: 7 g Ca, 1.5 g P, 1.5 g Na, 0.58 g Mg, 12 mg Cu, 73 mg Zn, 73 mg Mn, 87 mg Fe, 1.9 mg I, 0.38 mg Se, 11,600 IU Vitamin A, 1914 IU Vitamin D3, 116 mg Vitamin E, 2.2 mg Vitamin K, 2.2 mg Vitamin B1, 5.8 mg Vitamin B2, 4.4 mg Vitamin B6, 29 µg Vitamin B12, 29 mg nicotinic acid, 15 mg pantothenic acid, 0.87 mg folic acid, 116 µg biotin and 290 mg choline chloride (supplied by Bergophor, Kulmbach, Germany).

^c^ Analysed values for crude nutrients (mean of three determinations) and calculated values for energy and amino acid concentrations.

### 2.2 Sample Collection

Feed samples were collected at the beginning of the experiment and stored at −20°C for later analysis of crude nutrients. After 7 days, one piglet per box was killed, and after 5 weeks, the remaining two piglets per box were killed. At slaughter, faecal samples were collected from the rectum, stored at −20°C until they were lyophilised and milled for later analysis of titanium dioxide (TiO_2_) and crude nutrients. Segments of duodenum, proximal and mid‐jejunum, and proximal colon were collected, digesta of mid‐jejunum and proximal colon were sampled, and the pH was determined in the fresh proximal colon digesta. The small intestine segments were rinsed with 0.9% NaCl solution, cut into pieces and placed into 10% buffered formalin for later embedding in paraffin and Giemsa staining for examination of gut morphology (villi length, crypt depth), scoring of intestinal inflammation and lymphocyte infiltration (jejunum only) using a semi‐quantitative scoring system according to Day et al. (2008). In addition, the mucosa of the mid‐jejunum and proximal colon was collected by scraping with a cell scraper after removing digesta particles by rinsing with sterile 0.9% NaCl solution. All mucosa and digesta samples were snap‐frozen in liquid N_2_ and stored at −80°C until analysis.

### 2.3 Determination of Apparent Total Tract Digestibility of Nutrients

The apparent total tract digestibility (ATTD) was determined using TiO_2_ as an indigestible marker. In feed and faeces, concentrations of dry matter (DM), crude protein (CP), crude fibre (CF) and crude ash (CA) (Methods 3.1, 4.1.1, 6.1.1 and 8.1) (VDLUFA Verband Deutscher Landwirtschaftlicher Untersuchungs‐ und Forschungsanstalten 2013) were analysed. Owing to the low sample amount, lipids in feed and faeces were determined using the sulpho‐phospho‐vanillin reaction according to Zöllner and Kirsch (1962). Samples of feed (0.2 g) and lyophilised faeces (0.1 g) were extracted with 1 mL of hexane:isopropanol (3:2, vol/vol) by sonication for 30 min according to Hara and Radin (1978) after homogenisation with a TissueLyzer (Qiagen, Hilden, Germany) at 30 Hz for 3 min using 5 mm Stainless Steel Beads (Qiagen). After centrifugation (10 min, 15°C, 1900 × *g*), 150 μL of the upper fat‐containing phase was evaporated under N_2_ at 37°C. Subsequently, 2 mL of sulphuric acid was added, carefully whirled, kept at 105°C for 10 min and cooled down to RT. From this mixture, a 50 μL‐aliquot was combined with 1 mL of phosphoric acid/0.6% vanillin in distilled water (4:1, v/v), and incubated for 40 min at RT, and the absorption of the pink complex was determined spectrophotometrically at 530 nm. A standard curve was prepared with previously extracted lipids from feed and faeces, respectively. The concentration of TiO_2_ in diets and faeces was measured spectrophotometrically by the method of Brandt and Allam (1987) with modifications. Feed (1 g) or lyophilised faeces (100 mg) were combined with 3 or 1.5 g of Wieninger catalyst and 15 or 7.5 mL of sulphuric acid for acid hydrolysis. Afterwards, 2.5 mL of the sample was combined with 0.25 mL of 30% H_2_O_2_, incubated for 1 h, and the absorption was determined spectrophotometrically at 405 nm. The ATTD (%) was calculated as 100 − (% TiO_2_ in feed/% TiO_2_ in faeces × % nutrient in faeces/% nutrient in feed) × 100 according to Yin et al. (2000).

### 2.4 Histological Analysis of Gut Morphology

Longitudinal and cross‐sections of the collected small intestine segment samples were dehydrated in Tissue‐Tek 2000/VIP 5 Jr. (Sakura Finetek Germany GmbH, Staufen, Germany), embedded in paraffin, trimmed to 1.5 µm thickness with a microtome (Microm HM340 E, Microm international GmbH, Walldorf, Germany) and Giemsa stained (Giemsa‐Hollborn‐Original‐Stammlösung [0.5–10 mL aqua dest.], Dr K. Hollborn & Söhne, Leipzig, Germany). The sections were analysed and photographed by light microscopy (Nikon Eclipse 50i, Nikon Instruments Europe B.V., Düsseldorf, Germany). Villi length and crypt depth were processed and measured with Motic Images Plus 2.0 ML and ImageJ. In longitudinal sections, villi length and crypt depth were measured from 10 well‐oriented villi and crypts per sample, which were clearly not demolished and were met completely longitudinal. Villus length and crypt depth were related to the intestinal muscle layer diameter (*Stratum longitudinale* and *Stratum circulare* of the *Tunica muscularis*, measured in cross‐sections), which is an index for post‐mortal contraction of the muscle layer and produces a disturbant effect on villus length and crypt depth. Additionally, the ratio of villus height to crypt depth was calculated.

Histopathological scoring of intestinal inflammation and lymphocyte infiltration in Giemsa‐stained cross‐sections was based on Day et al. (2008). Per animal, four complete cross‐sections of duodenum, proximal and mid‐jejunum were assessed, and scores of one (normal mucosa) to four (strong pathological changes) were given for mucosa fibrosis, villus stunting and villous epithelial injury (see also footnotes of Table S5). In the proximal jejunum and mid‐jejunum, IEL and goblet cells were counted in 10 different villi per gut part per animal on a 200 µm length each. In the *lamina propria* of proximal jejunum and mid‐jejunum, numbers of lymphocytes, neutrophils and eosinophils were counted on five different sections (one high‐power field [0.237 mm^2^] each) per gut part and animal. In the duodenum, only the number of goblet cells was counted, because of random noise due to the presence of too many immune cells.

### 2.5 RNA Isolation and quantitative PCR Analysis in Gut Mucosa

Total RNA from mid‐jejunum and proximal colon mucosa samples (25–30 mg) was isolated using Trizol reagent (Thermo Fisher Scientific, Carlsbad, USA) according to the manufacturer's protocol. The cDNA synthesis and quantitative PCR (qPCR) analysis were performed according to Zeitz et al. (2016). The cDNA was diluted 1:2 with DNase/RNase‐free water and stored at −20°C until qPCR analysis. The characteristics of the gene‐specific primer pairs, which were designed using PRIMER3 and BLAST and obtained from Eurofins Genomics (Ebersberg, Germany), are listed in Table S1. Relative mRNA levels were calculated based on the comparative Ct method using the sample with the lowest Ct value as the reference Ct value as reported recently (Chiappisi et al. 2017). The qPCR data were normalised using multiple reference genes according to the approach from Vandesompele et al. (2002).

### 2.6 Determination of Short‐Chain Fatty Acids in the Colon Digesta

The total concentrations of the short‐chain fatty acids (SCFAs) were determined by gas chromatography (Clarus 580 GC system, Perkin Elmer, Waltham, USA) equipped with a flame‐ionisation detector and a split injector in 100 mg of colon digesta according to Fiesel et al. (2014). Fatty acids were separated on a polar capillary column (10 m free fatty acid phase, 0.32 mm internal diameter, 0.25 μm film thickness; Macherey and Nagel, Düren, Germany). Individual fatty acids were identified by comparing their retention times with those of individually purified standards. The fatty acid concentrations were calculated from the peak areas relative to the peak area of crotonic acid as the internal standard.

### 2.7 Analysis of Selected Bacterial Groups in the Colon by qPCR

Total genomic DNA was isolated from 200 mg of colon digesta with the QIAamp DNA Stool Mini Kit (Qiagen, Hilden, Germany) according to the instructions of the supplier. The absorbance at 260 nm (DNA concentration; 10–190 ng/µL) and the absorbance ratios of 260/280 nm (1.98 ± 0.09) were estimated from the optical density at 260 and 280 nm using an Infinite 200 M microplate reader and a NanoQuant Plate (Tecan), and DNA was stored at −20°C. The qPCR was carried out on a Rotorgene 2000 system (Corbett Research, Mortlake, Australia) using 2 μL DNA combined with 8 μL of a mixture composed of 5 μL KAPA SYBR FAST qPCR Universal Mastermix (Peqlab, Erlangen, Germany), 0.2 μL each of 10 μM forward and reverse primers and 2.6 μL DNase/RNase‐free water in 0.1 mL tubes (Ltf Labortechnik, Wasserburg, Germany). Specific primer pairs were obtained from Eurofins MWG Operon (Ebersberg, Germany) (Table S2). *Salmonella* spp. and *Escherichia coli* were detected only qualitatively, because copy numbers were too low to be reliably detected quantitatively. The prebiotic index was calculated as log copies of (*Bifidobacterium* spp. + *Lactobacillus* spp.)/(*Clostridium‐leptum‐*group + *Prevotella* spp.) instead of cell numbers of (Bifidobacteria + Lactobacilli)/(Bacteroides + Clostridia) as described in Chaikliang et al. (2015). The microbial balance index was calculated as log copies of (*Bifidobacterium* spp. + *Faecalibacterium prausnitzii*)/(*Prevotella* spp.) instead of cell numbers of (Bifidobacteria + *F. prausnitzii*‐group)/(Bacteroides − Porphyromonas − *Prevotella* group + enteric group bacteria) as described in Vaahtovuo et al. (2007).

### 2.8 Statistical Analysis

The data were analysed by two‐way ANOVA with the fixed factors treatment and experimental run and their interaction, using the general linear model procedure in Minitab statistical software (Release 13, Minitab Inc., State College, PA, USA). The Tukey method was used for multiple comparisons among means. The experimental unit was the box for performance data and the individual animal for laboratory analytical data. The data sets were checked for outliers. The histopathological scoring data set was analysed with the non‐parametric Kruskal–Wallis test.

## 3 Results

### 3.1 Growth Performance and ATTD of Nutrients

Final BW, daily BW gain, feed intake and feed:gain ratio of the piglets during the whole period and the individual periods did not differ across the three groups (Table 2). After 7 days of treatment, the ATTD of all crude nutrients with the exception of CA was not different across the groups (Table 3). The ATTD of CA was higher in the A100 group than in the control group (*p* < 0.05). The ATTD of CA did not differ between the A200 group and the other two groups. After 35 days, the ATTD of all crude nutrients was not different across the three groups.

**Table 2:** Performance of weaned piglets fed either a control diet (control group) or the control diet supplemented with algal β‐glucan at 100 (A100 group) or 200 g/t (A200 group) for 35 days.

				*p*‐value		
	Control group	A100 group	A200 group	Treatment	Run	Treatment × Run
Whole period (Days 1–35)						
Initial BW, kg	6.68 ± 0.91	6.71 ± 1.05	6.65 ± 1.05	0.99	0.58	0.96
Final BW, kg	23.5 ± 1.56	24.7 ± 1.74	24.7 ± 1.55	0.31	0.57	0.73
Daily BW gain, g	469 ± 36	497 ± 47	509 ± 41	0.25	0.61	0.85
Daily feed intake, g	651 ± 62	693 ± 65	710 ± 67	0.22	0.35	0.59
Feed:gain ratio	1.39 ± 0.04	1.40 ± 0.07	1.39 ± 0.06	0.95	0.24	0.33
Phase I (Days 1–16)						
Initial BW, kg	6.68 ± 0.44	6.71 ± 0.40	6.65 ± 0.54	0.99	0.52	0.95
Final BW, kg	11.0 ± 0.95	11.5 ± 0.94	11.1 ± 1.08	0.64	0.005	0.30
Daily BW gain, g	242 ± 38	259 ± 55	262 ± 54	0.37	0.001	0.35
Daily feed intake, g	275 ± 39	294 ± 47	300 ± 58	0.30	0.002	0.54
Feed:gain ratio	1.14 ± 0.10	1.15 ± 0.11	1.15 ± 0.07	0.95	0.21	0.42
Phase II (Days 17–35)						
Initial BW, kg	11.0 ± 0.95	11.5 ± 0.94	11.1 ± 1.08	0.64	0.005	0.30
Final BW, kg	23.5 ± 1.56	24.7 ± 1.74	24.7 ± 1.55	0.31	0.57	0.73
Daily BW gain, g	660 ± 58	697 ± 64	717 ± 53	0.25	0.18	1.00
Daily feed intake, g	1038 ± 104	1103 ± 99	1130 ± 87	0.22	0.90	0.64
Feed:gain ratio	1.57 ± 0.04	1.58 ± 0.08	1.58 ± 0.08	0.89	0.004	0.07

*Note:* Data are means ± SD for *n* = 8 boxes/group (control group) and *n* = 7 boxes/group (each A100 group and A200 group).

**Table 3:** ATTD of crude nutrients of weaned piglets fed either a control diet (control group) or the control diet supplemented with algal β‐glucan at 100 (A100 group) or 200 g/t (A200 group) for 7 or 35 days.

				*p*‐value		
	Control group	A100 group	A200 group	Treatment	Run	Treatment × Run
7 days						
Dry matter	94.6 ± 0.70	95.7 ± 0.98	95.1 ± 1.20	0.17	0.23	0.28
Crude ash	54.5 ± 1.76^b^	61.5 ± 5.27^a^	58.9 ± 6.03^a,b^	0.049	0.80	0.21
Organic matter	83.9 ± 2.05	86.4 ± 3.93	85.1 ± 3.38	0.40	0.59	0.34
Crude protein	71.3 ± 4.03	75.4 ± 8.16	76.4 ± 4.33	0.20	0.56	0.13
Crude fibre	63.0 ± 8.57	69.6 ± 12.81	67.5 ± 9.83	0.59	0.86	0.92
Lipids	73.3 ± 4.59	77.3 ± 7.21	75.1 ± 7.13	0.46	0.14	0.14
N‐free extract	92.1 ± 1.77	93.4 ± 1.95	91.3 ± 2.53	0.27	0.14	0.69
35 days						
Dry matter	95.5 ± 0.58	95.3 ± 0.47	95.7 ± 0.43	0.08	0.19	0.75
Crude ash	55.7 ± 4.19	56.9 ± 3.47	55.9 ± 1.94	0.72	0.03	0.89
Organic matter	87.3 ± 2.16	86.1 ± 1.73	87.2 ± 1.74	0.16	0.89	0.34
Crude protein	79.9 ± 3.77	79.8 ± 4.12	81.6 ± 3.97	0.43	0.69	0.34
Crude fibre	58.4 ± 10.13	51.7 ± 10.97	56.5 ± 8.49	0.14	0.96	0.23
Lipids	76.7 ± 3.20	75.2 ± 3.26	77.4 ± 2.36	0.10	0.06	0.77
N‐free extract	93.2 ± 1.31	92.1 ± 0.99	92.6 ± 1.22	0.08	0.61	0.80

*Note:* Data are means ± SD for *n* = 7–8 piglets per group (7 days) or *n* = 14–16 piglets per group (35 days). ^a,b^Means with no common superscript letter differ at *p* < 0.05.

### 3.2 Gut Morphology in Small Intestine

After 7 days of treatment, the villus length in the proximal jejunum was higher in the A100 group than in the control group and the A200 group (*p* < 0.05, Table 4), but did not differ between the A200 group and the control group. In addition, the villus length:crypt depth ratio was higher in the A100 group than in the A200 group, but it did not differ between the A100 group and the control group. In the duodenum and the mid‐jejunum, the gut morphology measures were not different across groups after 7 days (Table 4). After 35 days, gut morphology in the duodenum, mid‐jejunum and proximal jejunum was not different across groups (Table 4).

**Table 4:** Gut morphology of weaned piglets fed either a control diet (control group) or the control diet supplemented with algal β‐glucan at 100 (A100 group) or 200 g/t (A200 group) for 7 or 35 days.

				*p*‐value		
	Control group	A100 group	A200 group	Treatment	Run	Treatment × Run
7 days						
Duodenum						
Villus length, µm	1.55 ± 0.34	1.75 ± 0.39	1.53 ± 0.35	0.33	0.01	0.92
Crypt depth, µm	0.95 ± 0.14	1.03 ± 0.24	0.90 ± 0.18	0.15	0.00	0.10
Villus length:crypt depth	1.68 ± 0.56	1.71 ± 0.23	1.73 ± 0.36	0.97	0.97	0.41
Proximal jejunum						
Villus length, µm	1.90 ± 0.42^b^	3.20 ± 0.42^a^	2.02 ± 0.25^b^	< 0.001	0.05	0.21
Crypt depth, µm	0.80 ± 0.19	1.06 ± 0.22	0.93 ± 0.08	0.09	0.33	0.88
Villus length:crypt depth	2.43 ± 0.48^a,b^	3.16 ± 0.79^a^	2.18 ± 0.25^b^	0.03	0.60	0.53
Mid‐jejunum						
Villus length, µm	2.03 ± 0.48	2.29 ± 0.76	2.05 ± 0.31	0.64	0.14	0.37
Crypt depth, µm	0.93 ± 0.22	0.97 ± 0.17	0.88 ± 0.25	0.83	0.05	0.96
Villus length:crypt depth	2.21 ± 0.41	2.37 ± 0.67	2.39 ± 0.40	0.72	0.68	0.48
35 days						
Duodenum						
Villus length, µm	1.48 ± 0.34	1.70 ± 0.44	1.65 ± 0.39	0.85	0.00	0.41
Crypt depth, µm	0.69 ± 0.16	0.63 ± 0.13	0.63 ± 0.16	0.26	0.23	0.02
Villus length:crypt depth	2.23 ± 0.62	2.76 ± 0.72	2.65 ± 0.59	0.26	0.07	0.53
Proximal jejunum						
Villus length, µm	2.43 ± 0.78	2.43 ± 0.57	2.48 ± 0.67	0.85	0.02	0.61
Crypt depth, µm	0.72 ± 0.21	0.67 ± 0.18	0.71 ± 0.20	1.00	0.21	0.35
Villus length:crypt depth	3.44 ± 0.60	3.66 ± 0.55	3.54 ± 0.51	0.57	0.16	0.30
Mid‐jejunum						
Villus length, µm	2.12 ± 0.75	2.02 ± 0.57	1.72 ± 0.35	0.21	0.01	0.73
Crypt depth, µm	0.71 ± 0.24	0.71 ± 0.16	0.57 ± 0.11	0.14	0.36	0.33
Villus length:crypt depth	2.97 ± 0.34	2.82 ± 0.54	3.01 ± 0.42	0.53	0.00	0.26

*Note:* Data are means ± SD for *n* = 7–8 piglets per group (7 days) or *n* = 14–16 piglets per group (35 days). ^a,b^Means with no common superscript letter differ at *p* < 0.05. Villus and crypt length are given in µm/µm *Lamina muscularis* thickness to correct for post‐mortal contraction of the intestinal muscle layers.

### 3.3 Expression of Genes Involved in Inflammation, Gut Barrier and Nutrient Transport in the Jejunum and the Colon Mucosa

In the jejunum mucosa, the mRNA levels of genes involved in inflammation (*CCL2*, *ICAM1*, *IL8* and *TNFA*) and nutrient transport (*GLUT2*, *GLUT5*, *SGLT1* and *PEPT1*) were not different across the groups after 7 and 35 days of treatment (Table S3). The mRNA levels of TJ proteins of the gut barrier (*CLDN3*, *OCLN* and *ZO1*) in the jejunum mucosa were also not different across groups after 35 days (Table S3). After 7 days, the mRNA level of *OCLN* in the jejunum was lower in the A100 group than in the control group (*p* < 0.05, Table S3) but did not differ between the A200 group and the other two groups. The mRNA levels of *CLDN3* and *ZO1* in the jejunum mucosa after 7 days were also not different across groups.

In the colon mucosa, the mRNA levels of all genes involved in inflammation (*CCL2*, *ICAM1* and *IL8*) and gut barrier (*CLDN3*, *OCLN* and *ZO1*) with the exception of *OCLN* after 7 days and *ICAM1* after 35 days were not different across groups (Table S4). The mRNA level of *OCLN* in the colon mucosa after 7 days was lower in the A100 group than in the control group (*p* < 0.05) but did not differ between the A200 group and the other two groups. The mRNA level of *ICAM1* in the colon mucosa after 35 days was higher in the A100 group and the A200 group than in the control group (*p* < 0.05).

### 3.4 Counts of Gut‐Associated Immune Cells in the Small Intestine

In the proximal jejunum, the counts of IEL, goblet cells as well as lymphocytes, eosinophils and neutrophils in the *lamina propria* were not different across groups neither after 7 days nor after 35 days of treatment (Table 5). In the mid‐jejunum, the counts of goblet cells tended to be increased, and those of eosinophils in the *lamina propria* tended to be decreased in the A200 group compared to the control group after 7 days (*p* < 0.1, Table 5). After 35 days, the count of lymphocytes in the *lamina propria* of the mid‐jejunum was significantly higher in the A200 group than in the other two groups (*p* < 0.05). The goblet cell count in the mid‐jejunum tended to be increased, and the IEL count in the mid‐jejunum tended to be decreased in groups A100 and A200 compared to the control group after 35 days (*p* < 0.1).

**Table 5:** Counts of gut‐associated immune cells in the small intestine of weaned piglets fed either a control diet (control group) or the control diet supplemented with algal β‐glucan at 100 (A100 group) or 200 g/t (A200 group) for 7 or 35 days.

				*p*‐value		
	Control group	A100 group	A200 group	Treatment	Run	Treatment × Run
7 days						
Proximal jejunum						
IEL	7.37 ± 2.39	6.89 ± 2.39	5.59 ± 0.89	0.19	0.19	0.05
Goblet cells	0.52 ± 0.31	0.69 ± 0.33	0.91 ± 0.70	0.15	0.024	0.23
Lymphocytes in LP	21.37 ± 6.07	23.57 ± 7.32	24.52 ± 11.33	0.89	0.11	0.60
Eosinophils in LP	24.03 ± 8.53	26.49 ± 13.35	15.92 ± 8.87	0.29	0.003	0.82
Neutrophils in LP	0.57 ± 0.51	0.23 ± 0.23	0.24 ± 0.33	0.36	NA	NA
Mid‐jejunum						
IEL	7.83 ± 2.02	7.76 ± 1.86	6.80 ± 2.15	0.33	0.006	0.90
Goblet cells	0.63 ± 0.51	0.63 ± 0.59	1.17 ± 0.83	0.037	0.003	0.026
Lymphocytes in LP	25.05 ± 3.39	22.73 ± 1.37	21.57 ± 3.48	0.12	0.49	0.38
Eosinophils in LP	41.05 ± 5.74	31.87 ± 14.52	29.87 ± 9.89	0.07	0.94	0.038
Neutrophils in LP	0.45 ± 0.26	0.37 ± 0.27	0.26 ± 0.22	0.31	NA	NA
35 days						
Proximal jejunum						
IEL	23.97 ± 7.27	25.19 ± 4.31	25.10 ± 7.59	0.23	< 0.001	0.09
Goblet cells	1.47 ± 0.83	1.62 ± 0.77	1.54 ± 0.78	0.89	0.015	0.86
Lymphocytes in LP	27.64 ± 4.77	25.12 ± 4.18	26.20 ± 3.14	0.22	0.011	0.77
Eosinophils in LP	18.26 ± 6.14	16.58 ± 6.05	17.38 ± 3.40	0.72	0.47	0.93
Neutrophils in LP	0.19 ± 0.18	0.23 ± 0.21	0.25 ± 0.27	0.86	NA	NA
Mid‐jejunum						
IEL	25.11 ± 4.81	21.57 ± 5.11	23.43 ± 5.50	0.06	0.001	0.23
Goblet cells	1.33 ± 0.70	1.93 ± 0.94	2.14 ± 0.70	0.06	0.51	0.47
Lymphocytes in LP	24.79 ± 2.70^b^	24.87 ± 3.60^b^	29.51 ± 2.07^a^	< 0.001	0.11	0.033
Eosinophils in LP	36.09 ± 11.96	28.70 ± 7.77	38.73 ± 13.23	0.15	0.001	0.94
Neutrophils in LP	0.17 ± 0.18	0.29 ± 0.28	0.31 ± 0.27	0.36	NA	NA

*Note:* Data are means ± SD for *n* = 7–8 piglets per group (7 days) or *n* = 14–16 piglets per group (35 days). IEL = intraepithelial lymphocytes per 200 µm of villus length. Goblet cells: counted per 200 µm of villus length. LP = *Lamina propria*, all lymphocytes, eosinophils or neutrophils per high‐power field were counted. Statistical analysis with one‐way ANOVA for all parameters except for neutrophils, which were analysed with the non‐parametric Kruskal–Wallis test.

### 3.5 Mucosa Scoring for the Occurrence of Mucosa Fibrosis, Villous Stunting and Villous Epithelial Injury

Mucosa scoring for the occurrence of mucosa fibrosis, villous stunting and villous epithelial injury in the duodenum and proximal and mid‐jejunum showed no difference between groups after 7 days of treatment (Table S5). After 35 days, the villous stunting score in the proximal jejunum tended to be lower in the A100 group than in the other two groups (*p* < 0.1). The other mucosa scorings in the proximal jejunum and all mucosa scorings in the duodenum and the mid‐jejunum were not different across the groups after 35 days.

### 3.6 Concentrations of SCFA in the Colon Digesta

After 7 days of treatment, the concentrations and proportions of individual and total SCFA in the colon digesta did not differ across groups (Table 6). After 35 days, the proportion of acetate was higher and that of valerate was lower in the colon digesta of the A200 group than in the other two groups (*p* < 0.05). In addition, the concentration of valerate in the colon digesta was lower in the A200 group than in the other two groups after 35 days. The concentrations and proportions of the other individual and total SCFA were not different across groups.

**Table 6:** Abundance of bacterial groups in the colon digesta of weaned piglets fed either a control diet (control group) or the control diet supplemented with algal β‐glucan at 100 (A100 group) or 200 g/t (A200 group) for 7 or 35 days.

				*p*‐value		
	Control group	A100 group	A200 group	Treatment	Run	Treatment × Run
Log(copies)/g digesta						
7 days						
*Bifidobacterium* spp.	4.7 ± 0.69	4.5 ± 0.71	4.5 ± 0.55	0.85	0.55	0.62
*Clostridium‐leptum*‐group	7.4 ± 0.49	7.1 ± 0.53	6.9 ± 0.44	0.08	0.015	0.52
*Faecalibacterium prausnitzii*	6.6 ± 0.49^a^	6.5 ± 0.58^a,b^	6.1 ± 0.32^b^	0.037	0.052	0.018
*Lactobacillus* spp.	7.5 ± 0.37	7.4 ± 0.47	7.0 ± 0.39	0.08	0.85	0.58
*Prebiotic index* a	1.7 ± 0.17	1.7 ± 0.16	1.7 ± 0.09	0.93	0.012	0.63
35 days						
*Bifidobacterium* spp.	5.1 ± 0.33	4.8 ± 0.40	4.9 ± 0.44	0.22	0.99	0.92
*Clostridium‐leptum*‐group	6.9 ± 0.27	7.1 ± 0.36	7.0 ± 0.51	0.47	0.92	0.95
*Faecalibacterium prausnitzii*	6.6 ± 0.25	6.8 ± 0.32	6.7 ± 0.55	0.27	0.46	0.91
*Lactobacillus* spp.	6.9 ± 0.37	7.1 ± 0.50	6.9 ± 0.37	0.61	0.72	0.43
*Prevotella* spp.	6.9 ± 0.61^a^	6.9 ± 0.34^a^	6.4 ± 0.46^b^	0.028	0.19	0.78
*Prebiotic index* a	0.9 ± 0.04	0.8 ± 0.04	0.9 ± 0.06	0.22	0.99	0.45
*Microbial balance index* b	1.7 ± 0.14	1.7 ± 0.08	1.8 ± 0.13	0.11	0.53	0.66

*Note:* Data are means ± SD for *n* = 7–8 piglets per group (7 days) or *n* = 14–16 piglets per group (35 days). ^a,b^Means with no common superscript letter differ at *p* < 0.05.

^a^ Calculated as log copies of (*Bifidobacterium* spp. + *Lactobacillus* spp.)/(*Clostridium*‐*leptum*‐group + *Prevotella* spp.) instead of cell numbers of (Bifidobacteria + Lactobacilli)/(Bacteroides + Clostridia) as described in Chaikliang et al. (2015). In colon digesta of piglets slaughtered after 7 days of algal β‐glucan supplementation, *Prevotella* could not be reliably quantified and were not included in the calculation.

^b^ Calculated as log copies of (*Bifidobacterium* spp. + *Faecalibacterium prausnitzii*)/(*Prevotella* spp.) instead of cell numbers of (*Bifidobacteria* + *Faecalibacterium prausnitzii*‐group)/(Bacteroides − Porphyromonas − *Prevotella* group + enteric group bacteria) as described in Vaahtovuo et al. (2007).

### 3.7 Abundance of Selected Bacterial Groups in the Colon Digesta

After 7 days of treatment, the abundance of *F. prausnitzii* was significantly lower (*p* < 0.05) and that of *Lactobacillus* spp. and *Clostridium‐leptum*‐group tended to be lower (*p* < 0.1) in the A200 group than in the control group (Table 7). The abundance of *Bifidobacterium* spp. in the colon digesta was not different across groups. After 35 days, the abundance of *Prevotella* spp. was lower in the A200 group than in the other two groups. The abundance of *Bifidobacterium* spp., *Clostridium‐leptum*‐group, *F. prausnitzii* and *Lactobacillus* spp. in the colon digesta did not differ across groups. The prebiotic index after 7 and 35 days, and the microbial balance index after 35 days were not different across groups. *Salmonella* spp. were not detected in the colon digesta of the piglets.

**Table 7:** Short‐chain fatty acid (SCFA) concentrations in the colon digesta of weaned piglets fed either a control diet (control group) or the control diet supplemented with algal β‐glucan at 100 (A100 group) or 200 g/t (A200 group) for 7 or 35 days.

				*p*‐value		
	Control group	A100 group	A200 group	Treatment	Run	Treatment × Run
7 days						
SCFA, µmol/g digesta						
Acetate	58.3 ± 7.2	61.0 ± 11.5	55.3 ± 8.1	0.41	0.90	0.11
Propionate	26.8 ± 5.9	29.1 ± 4.2	23.8 ± 5.8	0.10	0.33	0.058
Butyrate	8.4 ± 2.9	9.6 ± 3.3	8.9 ± 3.6	0.81	0.38	0.60
Iso‐butyrate	0.8 ± 0.3	0.7 ± 0.4	0.7 ± 0.3	0.72	0.14	0.69
Valerate	1.8 ± 1.1	2.5 ± 1.3	1.7 ± 1.0	0.54	0.22	0.88
Iso‐valerate	1.1 ± 0.5	0.9 ± 0.5	1.1 ± 0.5	0.58	0.071	0.60
Total SCFA	97.2 ± 13.0	103.7 ± 17.2	91.4 ± 14.3	0.23	0.62	0.080
SCFA, mol/100 mol						
Acetate	60.3 ± 5.4	58.5 ± 2.7	60.6 ± 3.4	0.58	0.19	0.54
Propionate	27.3 ± 3.4	28.7 ± 5.1	25.9 ± 3.5	0.43	0.42	0.68
Butyrate	8.6 ± 2.1	9.0 ± 2.5	9.7 ± 3.8	0.68	0.45	0.62
Iso‐butyrate	0.84 ± 0.40	0.61 ± 0.34	0.77 ± 0.32	0.36	0.085	0.38
Valerate	1.75 ± 0.85	2.33 ± 1.06	1.87 ± 1.06	0.68	0.21	0.70
Iso‐valerate	1.21 ± 0.67	0.81 ± 0.47	1.18 ± 0.58	0.31	0.09	0.40
35 days						
SCFA, µmol/g digesta						
Acetate	71.2 ± 4.7	75.2 ± 7.6	74.3 ± 8.1	0.33	0.32	0.33
Propionate	36.2 ± 4.5	36.9 ± 6.0	34.0 ± 5.4	0.20	0.022	0.54
Butyrate	11.9 ± 2.1	12.9 ± 3.7	10.8 ± 1.8	0.20	0.84	0.09
Iso‐butyrate	0.4 ± 0.1	0.4 ± 0.1	0.5 ± 0.1	0.80	0.040	0.005
Valerate	2.0 ± 0.7^ab^	2.2 ± 1.1^a^	1.2 ± 0.4^b^	0.016	0.18	0.95
Iso‐valerate	0.6 ± 0.2	0.6 ± 0.1	0.6 ± 0.1	0.20	0.64	0.007
Total SCFA	122 ± 8.8	128 ± 14.7	121 ± 12.8	0.25	0.17	0.36
SCFA, mol/100 mol						
Acetate	58.3 ± 2.0^a^	58.8 ± 3.0^a^	61.3 ± 2.7^b^	0.014	0.21	0.86
Propionate	29.5 ± 2.5	28.7 ± 2.9	27.9 ± 2.9	0.24	0.027	0.47
Butyrate	9.7 ± 1.5	10.0 ± 2.2	8.9 ± 1.2	0.33	0.43	0.073
Iso‐butyrate	0.35 ± 0.12	0.35 ± 0.10	0.39 ± 0.12	0.30	0.010	0.016
Valerate	1.62 ± 0.56^a^	1.71 ± 0.72^a^	1.01 ± 0.31^b^	0.013	0.091	0.98
Iso‐valerate	0.49 ± 0.17	0.46 ± 0.12	0.53 ± 0.16	0.27	0.19	0.009

*Note:* Data are means ± SD for *n* = 7–8 piglets per group (7 days) or *n* = 14–16 piglets per group (35 days). ^a,b^Means with no common superscript letter differ at *p* < 0.05.

## 4 Discussion

The main finding of the present study was that supplementation with algae β‐glucans does not significantly affect growth performance and only marginally influences gut morphology, gut integrity and gut function in healthy weaned piglets. This conclusion is supported by observations such as the lack of differences in intestinal morphometric parameters in the small intestine and the expression of nutrient transporters (*GLUT2*, *GLUT5*, *SGLT1* and *PEPT1*) in the jejunum mucosa among the three groups of piglets at the end of the feeding trial. Additionally, the ATTD of all crude nutrients analysed showed no differences between the groups at the end of the feeding trial.

Contradictory findings regarding the effects of β‐glucan sources on growth performance and nutrient digestibility have been reported in other studies. For instance, dietary supplementation with the β‐glucan laminarin (150 and 300 g/t) has been shown to improve growth performance (e.g., gain‐to‐feed ratio) and increase ATTD of gross energy in weaned piglets (Walsh et al. 2012). Similarly, feeding diets supplemented with 300 g/t of laminarin increased the expression of nutrient transporters such as *SGLT1*, *GLUT1* and *GLUT2* in the ileum mucosa, as well as the ATTD of gross energy, DM, organic matter, CP, ash and neutral detergent fibre (Heim et al. 2014). Conversely, studies on another algal polysaccharide, fucoidan, have demonstrated no improvement in growth performance, nutrient and energy digestibility, or ileal expression of nutrient transporters when supplemented at similar doses (Walsh et al. 2012; Heim et al. 2014). These disparities can likely be attributed to the substantial differences in chemical structure and molecular weight between laminarin and fucoidan, which influence their physicochemical properties, such as solubility, viscosity, binding, bulking and fermentability (Singh and Kim 2021).

The structural differences between laminarin (β‐[1,3]‐linked glucans with β‐[1,6]‐linked side chains) and paramylon (a linear β‐glucan consisting solely of β‐[1,3]‐linkages), the main storage polysaccharide in *E. gracilis*, may also explain the lack of effect observed with algae β‐glucan supplementation in this study. Supporting this view, oat β‐glucans, which consist mainly of 1‐3 and 1‐4 β‐D‐glucans, have been shown to inhibit glucose transport *in vitro* in the everted rat small intestine (Zhang et al. 2016) and to suppress SGLT1‐ and GLUT2‐mediated glucose transport in IEC (Abbasi et al. 2016). Similarly, β‐glucans from *Saccharomyces cerevisiae* (50 mg/kg BW/day) reduced *SGLT1* expression in the intestinal mucosa of mice on high‐fat diets (Cao et al. 2016).

The marginal effects of algae β‐glucan supplementation on gut integrity in this study were assessed by examining intestinal expression of key TJ proteins, such as *CLDN3*, *OCLN* and *ZO1*, which are critical genes for gut barrier function. OCLN and CLDN3 are transmembrane TJ proteins forming a linear barrier at the apical–lateral membrane, while ZO1 is a scaffolding protein linking transmembrane TJ proteins to the intracellular cytoskeleton (Feldmann et al. 2005; González‐Mariscal et al. 2014). Although *OCLN* expression in the jejunum and colon mucosa was reduced in piglets fed the lower dose of algae β‐glucans compared to the control group after 7 days, the expression of all investigated TJ proteins, including *OCLN*, in the jejunum and colon mucosa remained unaffected by algae β‐glucan supplementation at the end of the trial. While the reasons for the observed reduction in *OCLN* expression after 7 days remain unclear, the overall findings suggest that algae β‐glucan supplementation did not alter gut integrity in these piglets.

Although the current findings indicate that algae β‐glucans neither improve nor impair gut integrity in healthy piglets, evidence from other studies suggests that β‐glucans may have beneficial effects under pathological conditions. For example, in *Salmonella*‐infected broilers, supplementation with *S. cerevisiae* β‐1,3/1,6‐glucans increased intestinal TJ protein gene expression, improved gut architecture (e.g., increased villus length) and elevated goblet cell numbers (Shao et al. 2013). Similarly, β‐1,3‐glucans from *E. gracilis* were reported to protect against ulcerative colitis via modulating the gut barrier, T‐cell immunity and gut microbiota in the dextran sulphate sodium‐induced colitis mouse model (Guo et al. 2025). In a piglet model with experimental *E. coli* infection, dietary supplementation with β‐1,3‐glucans from *E. gracilis* was observed to increase the expression of claudin and occludin in the jejunum (Kim et al. 2019). Transcellular permeability was also shown to be reduced with algal β‐glucan supplementation.

Given that the intestinal epithelium not only serves as a physical barrier to commensal and pathogenic microorganisms but also performs critical immune‐regulatory functions (Stadnyk 2002; Burkey et al. 2009; Neurath 2014; Peterson and Artis 2014), we analysed the effects of algae β‐glucan supplementation on various parameters of the gut‐associated immune system. An evaluation of the number of IEL, goblet cells, lymphocytes, eosinophils and neutrophils in the *lamina propria* of the jejunum revealed no effects in the proximal jejunum and only minor effects in the mid‐jejunum of the piglets. Among these, the number of goblet cells, which protect the gut barrier by producing mucin glycoproteins that coat the luminal surface of IEC, only tended to increase in the mid‐jejunum of piglets supplemented with the higher dose of algae β‐glucans. Similarly, an increase in goblet cell numbers was observed in *Salmonella*‐infected broilers supplemented with β‐glucans, where *Salmonella* infection alone reduced goblet cell numbers (Shao et al. 2013), and mucin protein encoding *MUC2* gene expression was upregulated in the jejunum and ileum of *E. coli*‐infected piglets (Kim et al. 2019).

While the number of lymphocytes in the *lamina propria* of the mid‐jejunum was significantly increased after 35 days of supplementation in piglets fed the higher dose of algae β‐glucans, the number of IEL—a lymphocyte fraction directly interacting with IEC by secreting cytokines and chemokines (Stadnyk 2002; Burkey et al. 2009; Neurath 2014)—only tended to decrease in piglets fed the lower dose. This contrasts with another study in weaned piglets, where IEL numbers increased following supplementation with yeast β‐glucans (Yang et al. 2016). Differences in outcomes between studies could reflect structural and physicochemical variations among β‐glucans. The inconsistent effects of algae β‐glucan supplementation on lymphocytes in different compartments of the gut mucosa (epithelium vs. *lamina propria*) leave the significance of these findings uncertain. However, mRNA expression of key proinflammatory genes such as *ICAM1*, *IL8* and *TNFA* in the jejunum mucosa showed no differences between groups, with a similar pattern observed in the colon mucosa, except for *ICAM1*, which was significantly upregulated in both algae β‐glucan‐supplemented groups. Since *ICAM1*, *IL8* and *TNFA* are typically coregulated by the nuclear factor kappa‐light‐chain‐enhancer of activated B‐cells (NF‐κB), the master regulator of inflammation (Lawrence 2009), the selective upregulation of *ICAM1* was unexpected. Additionally, *ICAM1* is usually stimulated by TNFα during infection or tissue injury (Lawrence 2009), yet *TNFA* expression was unaffected by algae β‐glucan supplementation. Given this discrepancy and the high standard deviation of *ICAM1* mRNA expression across groups, the observed induction may represent an artifact and should not be overemphasised. The relatively high standard deviation of *ICAM1* mRNA might result from its low expression level as indicated by the high Ct values as compared to those of other genes determined. In fact, a high variability in qPCR measurements can occur when a gene is expressed at low levels, as reactions near the detection limit are more susceptible to technical noise. Under these conditions, small fluctuations in template quantity, amplification efficiency or background signal can lead to disproportionately large differences in Ct values.

Overall, findings from proinflammatory gene expression and immune cell analysis suggest that algae β‐glucan supplementation did not induce an inflammatory response in IEC or intestinal immune cells. Consistent with this, eosinophil numbers in the *lamina propria*—typically elevated under inflammatory or infectious conditions (Yantiss 2015)—tended to decrease in the mid‐jejunum of piglets receiving the higher dose of algae β‐glucans for 7 days. Similarly, neutrophil numbers, which are recruited to inflamed intestinal tissue via macrophage‐derived cytokines to combat microbial invaders (Fournier and Parkos 2012), were unaffected, further suggesting no induction of an inflammatory response. Additionally, mucosal scoring for fibrosis, villus stunting and epithelial injury—hallmarks of gut mucosa damage—revealed minimal effects from algae β‐glucan supplementation across small intestine segments. Gut morphology analysis showed no impairment of villus length or villus length‐to‐crypt depth ratio in the duodenum and jejunum. On the contrary, a slight increase in villus length and villus length‐to‐crypt depth ratio was noted in the proximal jejunum after 7 days in piglets supplemented with algae β‐glucans. Thus, these findings indicate that the intestinal immune system and mucosal architecture of healthy piglets are unaffected by algae β‐glucan supplementation. This aligns with previous studies reporting no impact on immune parameters in healthy piglets supplemented with yeast β‐glucans (Hiss and Sauerwein 2003; Hester et al. 2012).

In contrast to these findings in healthy weaned piglets, substantial evidence supports the immunomodulatory benefits of β‐glucan supplementation under infectious conditions. For example, β‐glucans from *Paenibacillus polymyxa* reduced inflammatory responses to gram‐negative bacterial infection in weaned piglets by inhibiting proinflammatory cytokine production while enhancing anti‐inflammatory cytokine production (Hwang et al. 2008). Similarly, in *E. coli*‐challenged piglets, yeast β‐glucan supplementation elevated plasma levels of cytokines, including regulatory IL10 (Li et al. 2006). In another study, *E. coli‐*infected piglets fed *E. gracilis*‐derived β‐glucans showed an increase in the expression of Dectin‐1, a pattern recognition receptor that is involved in the immune response to β‐glucans in the jejunum and ileum of (Kim et al. 2019). In the same study, an increase of CD4^+^ and CD8^+^ T‐lymphocytes was also observed in the blood of β‐glucan‐supplemented piglets with *E. coli* infection, which may be related to the lower febrile response and diarrhoeal disease observed in the piglets fed the *E. gracilis*‐derived β‐glucans (Kim et al. 2019). Furthermore, treating porcine peripheral blood mononuclear cells with β‐glucans of various origins (fungi, yeast, seaweed, bacteria or algae) and structures (linear or branched; soluble, gel or particulate) demonstrated that paramylon from *E. gracilis* enhanced reactive oxygen species production and cytokine release (Sonck et al. 2010).

Several studies have demonstrated that the supplementation of β‐glucans and algal polysaccharides modifies gut microbial community structure and bacterial fermentation in weaned piglets (Sweeney et al. 2011; Murphy et al. 2013; Walsh et al. 2013; Zhou et al. 2013; Luo et al. 2019), albeit with inconsistent results. While Sweeney et al. (2011) and Luo et al. (2019) observed a beneficial increase in gut‐protective *Lactobacillus* and/or *Bifidobacterium* populations in response to supplementation with the algal polysaccharide fucoidan (240 g/t diet) and β‐glucan from *Agrobacterium* sp. ZX09 (50–100 g/t diet), respectively, two other studies found no effect on these bacteria following supplementation with β‐glucans from *S. cerevisiae* (100 g/t diet) or laminarin from *Laminaria digitata* (300 g/t diet). Instead, both studies reported a reduction in pathogenic *E. coli* numbers (Murphy et al. 2013; Zhou et al. 2013). Similarly, a decrease in *E. coli* population was noted after supplementation with β‐glucan from *Agrobacterium* sp. ZX09 (Luo et al. 2019).

Consistent with these mixed findings, supplementation with algal β‐glucans to weaned piglets in the present study did not alter the populations of *Bifidobacterium* spp. and *Lactobacillus* spp. at Days 7 and 35, although a reduction in *F. prausnitzii* population was observed in the colon at Day 7. Since *F. prausnitzii* is recognised as an indicator of intestinal health in humans (Miquel et al. 2013) and its abundance is typically reduced in individuals with intestinal and metabolic disorders (Sokol et al. 2008; Neish 2009), this reduction represents an undesirable effect of supplementation with algae β‐glucans. However, given that this effect was absent at Day 35 and the prebiotic and microbial indices (according to Vaahtovuo et al. 2007) remained unchanged at the end of the trial, it suggests that algal β‐glucan supplementation exerts no significant impact on gut microbial community structure. The transient reduction in *F. prausnitzii* abundance in the colon of piglets observed at Day 7 of supplementation likely reflects the instability of the post‐weaning gut microbiota, a period characterised by profound shifts in microbial composition and reduced resilience to dietary perturbations. Early after weaning, the intestinal ecosystem is highly sensitive to stress and substrate changes explaining that marked shifts in microbial community structure can occur within this period. Albeit being speculative, it is possible that the algal β‐glucans caused a selective enrichment of certain bacterial taxa in the gut of the piglets, potentially inducing temporary competitive dynamics unfavourable to *F. prausnitzii* colonisation. By 35 days post‐weaning, however, the piglet microbiota typically regains structural stability—a fact that has presumably allowed *F. prausnitzii* to reestablish its ecological niche. The observed microbial colonisation pattern of the piglets indicates that the initial decline represents a short‐term adaptive response of a developing microbiome rather than a lasting detrimental effect of algal β‐glucan supplementation.

Furthermore, there were only minor shifts in the SCFA profile of colon digesta, such as slight increases in the proportions of acetate and valerate. These inconsistent effects of β‐glucans from various sources on gut microbiota and microbial fermentation activity are likely attributable to the divergent physicochemical properties arising from differences in their structure and molecular weight. As mentioned above, paramylon consists almost entirely of linear β‐1,3‐linked glucose residues, enabling these chains to align closely and form highly ordered, crystalline structures. In contrast, branched β‐1,3/β‐1,6 glucans from yeasts disrupt chain packing and generate a more amorphous, open structure that can be more readily accessed by enzymes. Consequently, paramylon is comparatively resistant to microbial enzymatic degradation, making it less fermentable than more branched or soluble β‐glucans that provide greater accessibility to the gut microbiota.

In conclusion, this study shows that algal β‐glucan supplementation in healthy weaned piglets has no effect on growth performance and only marginally influences gut morphology, gut integrity, gut‐associated immune function, digestibility and gut microbial community structure. The marginal influence of algal β‐glucans in the gut of healthy piglets in the present study might be explained by the fact that their mucosal immune system is stable, minimally activated and supported by an intact epithelial barrier. Under such homeostatic conditions, there is neither an increased need for immunomodulatory stimuli nor notable dysregulation in microbial colonisation that β‐glucans could correct. In contrast, in diseased or pathogen‐challenged piglets, the immune system is activated, epithelial integrity may be compromised and microbial balance is often disturbed. Under these stress conditions, β‐glucans can express their immunomodulatory properties more effectively, for example, by enhancing innate defence mechanisms, supporting barrier function or modulating microbial colonisation patterns. Thus, future research should investigate whether algal β‐glucan supplementation might yield beneficial outcomes in pathogen‐challenged weaned piglets.

## Ethics Statement

The experimental protocol of the study reported has been approved by the Animal Welfare Officer of the Justus Liebig University Giessen (approval no.: JLU 602_M). All experimental procedures described followed established guidelines for the care and handling of laboratory animals.

## Conflicts of Interest

R.W. and J.K.H. are employed by Evonik Industries. Their role was limited to the review of the manuscript. The other authors have declared no real or perceived conflicts of interest.

## Animal Welfare Statement

The authors confirm that the ethical policies of the journal, as noted on the journal's author guidelines page, have been adhered to and the appropriate ethical review committee approval has been received. The authors confirm that they have followed EU standards for the protection of animals used for scientific purposes (and feed legislation, if appropriate).

## Supporting information

Supporting File

## Data Availability

The data that support the findings of this study are available from the corresponding author upon reasonable request.
