# Supplementary material for: Effects of Algae β‐Glucan Supplementation on the Performance and Intestinal Immune System of Healthy Weaned Piglets
Source: J Anim Physiol Anim Nutr (Berl). 2026 May 20;110(5):642–54. doi: 10.1111/jpn.70077 (PMC13576544; doi:10.1111/jpn.70077)
Supplement: Supplementary file 1 [file JPN-110-642-s001.docx]

**Supplemental Table S1**	Characteristics of the primer pairs for gene expression analysis^*^

| Gene | Forward primer (from 5´ to 3´)  Reverse primer (from 5´ to 3´) | PCR product, bp | Annealing Temperature, °C | PCR efficiency | NCBI GenBank |
| --- | --- | --- | --- | --- | --- |
| Reference genes^†^ | |  |  |  |  |
| *ACTB* | GACATCCGCAAGGACCTCTA ACATCTGCTGGAAGGTGGAC | 205 | 60 | 1.91 | XM_003124280.3 |
| *ATP5G1* | CAGTCACCTTGAGCCGGGCGA  TAGCGCCCCGGTGGTTTGC | 94 | 64 | 2.07 | NM_001025218.2 |
| *B2MI* | CAAGATAGTTAAGTGGGATCGAGAC  TCAGAAATCGTATTGATGTTACCA | 161 | 58 | 1.80 | NM_213978 |
| *GPI* | CACGAGCACCGCTCTGACCT  CCACTCCGGACACGCTTGCA | 365 | 64 | 1.93 | NM_214330.1 |
| *RPS9* | GTCGCAAGACTTATGTGACC  AGCTTAAAGACCTGGGTCTG | 327 | 60 | 1.91 | XM_005664825.1 |
| *SDHA* | CTACGCCCCCGTCGCAAAGG  AGTTTGCCCCCAGGCGGTTG | 380 | 64 | 1.82 | DQ402993 |
| Target genes | |  |  |  |  |
| *IL8* | ACTTCCAAACTGGCTGTTGC  GGAATGCGTATTTATGCACTGG | 120 | 60 | 1.95 | NM_213867.1 |
| *TNF* | CATGAGCACTGAGAGCATGA  CGATAACTTCGAAGTGCAGT | 170 | 60 | 1.92 | NM_214022.1 |
| *CCL2* | GGTCCTTGCCCAGCCAGATGC  CTGCACAGATCTCCTTGCCCGC | 170 |  | 1.84 | NM_214214.1 |
| *ICAM1* | CGGTGGCAGCCGTGGCTATC  TTGATGCAGCCCCGCTCGTC | 208 | 60 | 1.88 | NM_213816.1 |
| *CLDN3* | TACGACCGCAAGGACTACG  TAGCATCTGGGTGGACTGG | 122 | 58 | 1.96 | NM_001160075.1 |
| *OCLN* | GAGTACATGGCTGCTGCTGA  TTTGCTCTTCAACTGCTTGC | 102 | 60 | 2.08 | NM_001163647.2 |
| *ZO1* | AAGCCCTAAGTTCAATCACAATCT  ATCAAACTCAGGAGGCGGC | 130 | 57 | 1.98 | XM_003353439.2 |
| *SLC2A2* | GCTGGATGGGGAAGCCAAAGCA  AGAGCGTCGCCCTGCCTTCT | 355 | 61 | 2.09 | NM_001097417.1 |
| *SLC2A5* | CTGACACTGGTGCTTGCTTT  TTCGCTCATGTATTCCCCGA | 156 | 61 | 1.92 | EU012359.2 |
| *SLC5A1* | GTGGCGGACAGTAGTGAACA AGAAGGCAGGATTTCAGGCA | 89 | 57 | 1.99 | NM_001164021.1 |
| *SLC15A1* | CAGACTTCGACCACAACGGA TTATCCCGCCAGTACCCAGA | 99 | 59 | 1.98 | NM_214347.1 |

^*^ACTB, beta-actin; ATP5G1, ATP synthase lipid-binding protein; B2MI, Beta-2-microglobulin; GPI, glucose-6-phosphate isomerase; RPS9, ribosomal protein S9; SDHA, succinate dehydrogenase; IL1B, interleukin 1 beta; IL8, interleukin 8; TNF, tumor necrosis factor; CCL2, C-C motif chemokine ligand 2; ICAM1, intercellular adhesion molecule 1; CLDN3, claudin 3; OCLN, occluding; ZO-1, zona occludens 1 (tight junction protein 1); SLC2A2: solute carrier family 2 member 2 (facilitated glucose transporter) (Glut2); SLC2A5: solute carrier family 2 member 5 (facilitated glucose transporter) (Glut5); SLC5A1, solute carrier family 5 member 1 (SGLT1); SLC15A1, solute carrier family 15 member 1 (oligopeptide transporter) (PEPT1).

^†^GeNorm normalization factor was calculated as the geometric mean of expression data (relative quantities) of the two most stable (ACTB, RPS9 in jejunum) or four most stable (ATP5G1, GPI, RPS9, SDHA in colon at day 7; ACTB, ATP5G1, GPI, SDHA in colon at day 35).

**Supplemental Table S2**	Characteristics of the primer pairs to detect bacterial groups

| Organism | Gene | Forward primer (from 5´ to 3´)  Reverse primer (from 5´ to 3´) | PCR product size, bp | Annealing temperature, °C | Primer efficiency | Reference |
| --- | --- | --- | --- | --- | --- | --- |
| *Bifidobacterium spp.* | 16S rDNA | TCGCGTC(C/T)GGTGTGAAAG  CCACATCCAGC(A/G)TCCAC | 243 | 56 | 1.97 | Fiesel et al., 2014 |
| *Clostridium-leptum-*group | 16S rDNA | GCACAAGCAGTGGAGT  CTTCCTCCGTTTTGTCAA | 246 | 49 | 2.04 | Matsuki et al., 2004 |
| *E. coli* | 16S rDNA | GTTAATACCTTTGCTCATTGA  AACAGGATTAGATACCCTGGT | 340 | 51 | 2.02 | Malinen et al., 2003 |
| *F. prausnitzii* | 16S rDNA | GGAGGAAGAAGGTCTTCGG  AATTCCGCCTACCTCTGCACT | 248 | 57 | 2.09 | Vital et al., 2013 |
| *Lactobacillus spp.* | 16S rDNA | AGCAGTAGGGAATCTTCCA  CACCGCTACACATGGAG | 341 | 62 | 2.02 | Fiesel et al., 2014 |
| *Prevotella spp.* | 16S rDNA | ACCAGCCAAGTAGCGTGCA  TGGACCTTCCGTATTACCGC | 152 | 59 | 1.98 | Martin et al., 2002 |
| *Salmonella spp.* | ompC | ATCGCTGACTTATGCAATCG  CGGGTTGCGTTATAGGTCTG | 204 | 57 | 2.07 | Cossi et al., 2014 |

**Supplemental Table S3**	Relative mRNA abundance of genes involved in inflammation, gut barrier and nutrient transport in the jejunum mucosa of weaned piglets fed either a control diet (control group) or the control diet supplemented with algal β-glucan at 100 (A100 group) or 200 g/t (A200 group) for 7 days (n = 7-8 piglets per group) or 35 days (n = 14-16 piglets per group)

|  |  |  |  |  |  |  |  |  |  | *P*-value | | |
| --- | --- | --- | --- | --- | --- | --- | --- | --- | --- | --- | --- | --- |
|  | Control group | | | A100 group | | | A200 group | | | Treatment | Run | Treatment x Run |
| *Day 7* |  |  |  |  |  |  |  |  |  |  |  |  |
| *IL8* | 1.00 | ± | 0.70 | 0.62 | ± | 0.34 | 0.72 | ± | 0.45 | 0.35 | 0.98 | 0.25 |
| *TNFA* | 1.00 | ± | 0.77 | 0.52 | ± | 0.14 | 0.56 | ± | 0.26 | 0.40 | 0.55 | 0.84 |
| *ICAM1* | 1.00 | ± | 0.74 | 0.57 | ± | 0.37 | 0.77 | ± | 0.34 | 0.053 | 0.006 | 0.37 |
| *CCL2* | 1.00 | ± | 0.63 | 0.63 | ± | 0.37 | 0.90 | ± | 0.50 | 0.55 | 0.58 | 0.62 |
| *CLDN3* | 1.00 | ± | 0.16 | 0.68 | ± | 0.33 | 0.95 | ± | 0.25 | 0.23 | 0.87 | 0.57 |
| *OCLN* | 1.00 | ± | 0.46^a^ | 0.47 | ± | 0.16^b^ | 0.69 | ± | 0.27^ab^ | 0.025 | 0.003 | 0.08 |
| *ZO1* | 1.00 | ± | 0.32 | 0.97 | ± | 0.13 | 0.93 | ± | 0.13 | 0.95 | 0.846 | 0.60 |
| *GLUT2* | 1.00 | ± | 0.27 | 0.74 | ± | 0.21 | 0.79 | ± | 0.36 | 0.22 | 0.11 | 0.84 |
| *GLUT5* | 1.00 | ± | 0.34 | 0.72 | ± | 0.17 | 0.99 | ± | 0.77 | 0.42 | 0.052 | 0.33 |
| *SGLT1* | 1.00 | ± | 0.57 | 0.61 | ± | 0.14 | 0.50 | ± | 0.29 | 0.30 | 0.229 | 0.36 |
| *PEPT1* | 1.00 | ± | 0.46 | 0.86 | ± | 0.19 | 0.84 | ± | 0.28 | 0.95 | 0.77 | 0.26 |
|  |  |  |  |  |  |  |  |  |  |  |  |  |
| *Day 35* |  |  |  |  |  |  |  |  |  |  |  |  |
| *IL8* | 1.00 | ± | 0.77 | 1.00 | ± | 0.75 | 0.56 | ± | 0.37 | 0.33 | 0.49 | 0.87 |
| *TNF* | 1.00 | ± | 1.06 | 0.83 | ± | 0.73 | 1.90 | ± | 2.16 | 0.43 | 0.023 | 0.07 |
| *ICAM1* | 1.00 | ± | 0.28 | 1.60 | ± | 0.91 | 1.42 | ± | 0.87 | 0.42 | 0.86 | 0.03 |
| *CCL2* | 1.00 | ± | 1.28 | 0.24 | ± | 0.17 | 0.64 | ± | 0.68 | 0.40 | 0.097 | 0.61 |
| *CLDN3* | 1.00 | ± | 0.23 | 1.06 | ± | 0.23 | 0.95 | ± | 0.23 | 0.069 | 0.000 | 0.93 |
| *OCLN* | 1.00 | ± | 0.65 | 0.79 | ± | 0.39 | 0.99 | ± | 0.54 | 0.66 | 0.000 | 0.82 |
| *ZO1* | 1.00 | ± | 0.63 | 1.06 | ± | 0.76 | 1.06 | ± | 0.71 | 0.21 | 0.000 | 0.66 |
| *GLUT2* | 1.00 | ± | 0.47 | 1.52 | ± | 1.00 | 0.99 | ± | 0.65 | 0.078 | 0.018 | 0.26 |
| *GLUT5* | 1.00 | ± | 0.63 | 0.82 | ± | 0.63 | 0.77 | ± | 0.36 | 0.29 | 0.000 | 0.26 |
| *SGLT1* | 1.00 | ± | 0.49 | 1.13 | ± | 0.57 | 0.99 | ± | 0.65 | 0.24 | 0.000 | 0.64 |
| *PEPT1* | 1.00 | ± | 0.68 | 1.02 | ± | 0.62 | 0.96 | ± | 0.62 | 0.55 | 0.000 | 0.94 |

Data are means ± SD for n = 7-8 piglets per group (7 days) or n = 14-16 piglets per group (35 days). ^a,b^Means with no common superscript letter differ at *P* < 0.05.

**Supplemental Table S4**	Relative mRNA abundance of genes involved in inflammation, gut barrier and nutrient transport in the colon mucosa of weaned piglets fed either a control diet (control group) or the control diet supplemented with algal β-glucan at 100 (A100 group) or 200 g/t (A200 group) for 7 days (n = 7-8 piglets per group) or 35 days (n = 14-16 piglets per group)

|  |  |  |  |  |  |  |  |  |  | *P*-value | | |
| --- | --- | --- | --- | --- | --- | --- | --- | --- | --- | --- | --- | --- |
|  | Control group | | | A100 group | | | A200 group | | | Treatment | Run | Treatment x Run |
| Day 7 |  |  |  |  |  |  |  |  |  |  |  |  |
| *IL8* | 1.00 | ± | 0.69 | 0.80 | ± | 0.45 | 0.65 | ± | 0.46 | 0.54 | 0.72 | 0.20 |
| *ICAM1* | 1.00 | ± | 0.21 | 1.45 | ± | 0.63 | 1.40 | ± | 0.51 | 0.37 | 0.69 | 0.51 |
| *CCL2* | 1.00 | ± | 0.54 | 0.97 | ± | 1.02 | 0.79 | ± | 0.50 | 0.94 | 0.69 | 0.29 |
| *CLDN3* | 1.00 | ± | 0.20 | 1.14 | ± | 0.48 | 1.01 | ± | 0.36 | 0.76 | 0.014 | 0.058 |
| *OCLN* | 1.00 | ± | 0.51^a^ | 0.58 | ± | 0.19^b^ | 0.71 | ± | 0.13^ab^ | 0.054 | 0.006 | 0.007 |
| *ZO1* | 1.00 | ± | 0.45 | 0.81 | ± | 0.27 | 0.88 | ± | 0.28 | 0.81 | 0.028 | 0.60 |
| Day 35 |  |  |  |  |  |  |  |  |  |  |  |  |
| *IL8* | 1.00 | ± | 0.67 | 0.38 | ± | 0.33 | 1.08 | ± | 0.57 | 0.19 | 0.71 | 0.49 |
| *ICAM1* | 1.00 | ± | 0.89^a^ | 1.34 | ± | 1.38^b^ | 1.82 | ± | 1.20^b^ | 0.022 | 0.069 | 0.008 |
| *CCL2* | 1.00 | ± | 0.88 | 0.35 | ± | 0.33 | 0.87 | ± | 0.54 | 0.17 | 0.89 | 0.24 |
| *CLDN3* | 1.00 | ± | 0.57 | 0.66 | ± | 0.23 | 0.85 | ± | 0.38 | 0.51 | 0.74 | 0.45 |
| *OCLN* | 1.00 | ± | 0.59 | 0.50 | ± | 0.39 | 0.74 | ± | 0.42 | 0.16 | 0.95 | 0.61 |
| *ZO1* | 1.00 | ± | 0.95 | 0.37 | ± | 0.24 | 0.62 | ± | 0.40 | 0.38 | 0.39 | 0.77 |

Data are means ± SD for n = 7-8 piglets per group (7 days) or n = 14-16 piglets per group (35 days). ^a,b^Means with no common superscript letter differ at *P* < 0.05.

**Supplemental Table S5**	Mucosa scoring for the occurrence of mucosa fibrosis, villous stunting and villous epithelial injury in the small intestine of weaned piglets fed either a control diet (control group) or the control diet supplemented with algal β-glucan at 100 (A100 group) or 200 g/t (A200 group) for 7 or 35 days^1^

|  | Control group | | | A100 group | | | A200 group | | | *P*-value |
| --- | --- | --- | --- | --- | --- | --- | --- | --- | --- | --- |
| *7 days* |  |  |  |  |  |  |  |  |  |  |
| *Duodenum* |  |  |  |  |  |  |  |  |  |  |
| Mucosa fibrosis score ^2^ | 1.25 | ± | 0.46 | 1.71 | ± | 0.49 | 1.43 | ± | 0.53 | 0.21 |
| Villous stunting score ^3^ | 1.75 | ± | 0.71 | 1.71 | ± | 0.49 | 2.00 | ± | 0.82 | 0.73 |
| Villous epithelial injury score ^4^ | 1.00 | ± | 0.00 | 1.14 | ± | 0.38 | 1.29 | ± | 0.49 | 0.29 |
| *Proximal jejunum* |  |  |  |  |  |  |  |  |  |  |
| Mucosa fibrosis score | 1.14 | ± | 0.46 | 1.00 | ± | 0.00 | 1.00 | ± | 0.00 | 0.16 |
| Villous stunting score | 1.43 | ± | 0.74 | 1.71 | ± | 0.49 | 2.00 | ± | 0.00 | 0.29 |
| Villous epithelial injury score | 1.14 | ± | 0.74 | 1.29 | ± | 0.49 | 1.29 | ± | 0.49 | 1.00 |
| *Mid jejunum* |  |  |  |  |  |  |  |  |  |  |
| Mucosa fibrosis score | 1.13 | ± | 0.35 | 1.29 | ± | 0.49 | 1.29 | ± | 0.49 | 0.70 |
| Villous stunting score | 1.50 | ± | 0.53 | 1.57 | ± | 0.53 | 1.71 | ± | 0.49 | 0.71 |
| Villous epithelial injury score | 1.00 | ± | 0.00 | 1.29 | ± | 0.49 | 1.00 | ± | 0.00 | 0.11 |
|  |  |  |  |  |  |  |  |  |  |  |
| *35 days* |  |  |  |  |  |  |  |  |  |  |
| *Duodenum* |  |  |  |  |  |  |  |  |  |  |
| Mucosa fibrosis score | 1.63 | ± | 0.62 | 1.43 | ± | 0.51 | 1.57 | ± | 0.51 | 0.65 |
| Villous stunting score | 1.75 | ± | 0.58 | 1.79 | ± | 0.58 | 1.71 | ± | 0.61 | 0.94 |
| Villous epithelial injury score | 1.63 | ± | 0.72 | 1.29 | ± | 0.47 | 1.43 | ± | 0.76 | 0.37 |
| *Proximal jejunum* |  |  |  |  |  |  |  |  |  |  |
| Mucosa fibrosis score | 1.31 | ± | 0.48 | 1.36 | ± | 0.50 | 1.31 | ± | 0.48 | 0.96 |
| Villous stunting score | 1.69 | ± | 0.48 | 1.29 | ± | 0.47 | 1.69 | ± | 0.48 | 0.047^5^ |
| Villous epithelial injury score | 1.25 | ± | 0.45 | 1.50 | ± | 0.52 | 1.23 | ± | 0.44 | 0.24 |
| *Mid jejunum* |  |  |  |  |  |  |  |  |  |  |
| Mucosa fibrosis score | 1.31 | ± | 0.48 | 1.29 | ± | 0.47 | 1.21 | ± | 0.43 | 0.83 |
| Villous stunting score | 1.38 | ± | 0.50 | 1.50 | ± | 0.52 | 1.50 | ± | 0.52 | 0.73 |
| Villous epithelial injury score | 1.63 | ± | 0.72 | 1.50 | ± | 0.85 | 1.43 | ± | 0.76 | 0.58 |

^1^The data set was analysed with the non-parametric Kruskal Wallis test. Data are means ± SD for n = 7-8 piglets per group (7 days) or n = 14-16 piglets per group (35 days).

^2^Mucosa fibrosis score: 1 = Normal mucosa (narrow band of stroma, up to 1 to 2 fibroblasts in width, separates crypts), 2 = Mild mucosal fibrosis (crypts separated by a band of stroma, up to 5 fibroblasts in width), 3 = Moderate mucosal fibrosis (crypts separated by a band of stroma, up to 10 fibroblasts in width. Crypts may vary in width, some being atrophic), 4 = Marked mucosal fibrosis (crypts separated by an extensive area of collagenous matrix, >10 fibroblasts in width. Crypts may be atrophic or lost and replaced by fibrotic matrix).

^3^Villous stunting score: 1 = Normal mucosa (long, slender uniform villi when sectioned longitudinally), 2= Mild villous stunting (villi reduced to approximately 75% of normal length; some may be increased in width and non-uniform), 3 = Moderate villous stunting (villi reduced to approximately 50% of normal length (stunted); most are increased in width and some may be fused), 4 = Marked villous stunting (villi reduced to <25% of normal length and often fused; intestinal surface may be flat in severe cases)

^4^Villous epithelial injury score: 1 = Normal mucosa (single layer of columnar epithelium), 2 = Mild villous epithelial injury (Attenuation, degeneration, vacuolation or separation of focal areas of superficial epithelium), 3 = Moderate villous epithelial injury (more marked degenerative changes, with focal loss of some epithelium), 4 = Marked villous epithelial injury (widespread ulceration of surface epithelium)

^5^No significant differences in the post hoc test (Mann-Whitney Test).
